# A Case of Torsion of Gravid Uterus Caused by Leiomyoma

**DOI:** 10.1155/2011/206418

**Published:** 2011-11-20

**Authors:** Gururaj Deshpande, Rajesh Kaul, Manjuladevi P.

**Affiliations:** Department of Obstetrics and Gynecology, Kamineni Institute of Medical Sciences, Nalgonda District, Andhra Pradesh State, Narketpally 508254, India

## Abstract

Uterine torsion during pregnancy is only sporadically reported in the literature. Here we present a case of leiomyoma causing uterine torsion in pregnancy and review the literature on etiology, diagnosis, and management. A 25-years-old primigravida with leiomyoma complicating pregnancy was admitted in our hospital with abdominal pain and uterine tenderness. She underwent emergency LSCS (lower segment cesarean section) for fetal bradycardia. Intraoperatively, the uterus was rotated 180 degrees left to right. Inadvertent incision on the posterior wall was avoided by proper delineation of anatomy. Torsion was corrected by exteriorization of leiomyoma and uterus, and lower segment cesarean was carried out safely. Prompt recognition and management of this condition is necessary for better maternal and fetal outcome.

## 1. Introduction

Rotation of the pregnant uterus is common during pregnancy but rarely exceeds 45 degrees and is most often to the right [1–3]. When the uterus rotates on itself, its blood supply decreases, which is called uterine torsion. Uterine venous obstruction causes increased pressure in placental cotyledons leading to abruption and fetal distress. When it progresses to uterine artery obstruction placental perfusion reduces which can lead to fetal demise. Uterine leiomyoma complicate 1.4% of pregnancies. Myoma complication depends on their location and size. These include red or carneous degeneration presenting with fever and leucocytosis and torsion of subserosal myomas. In this case, one-sided large intramural myoma caused uterus to rotate 180 degrees.

## 2. Case Presentation

**Figure 1:**
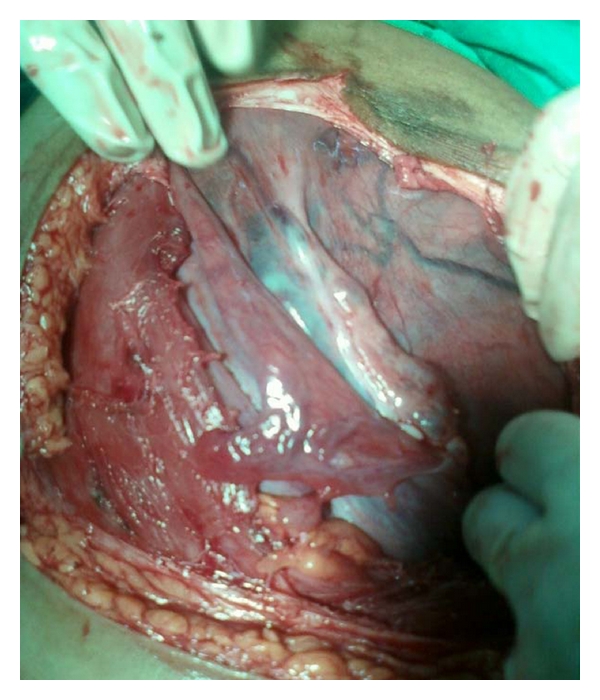
Posterior wall uterus with left adnexa turned to right.

**Figure 2:**
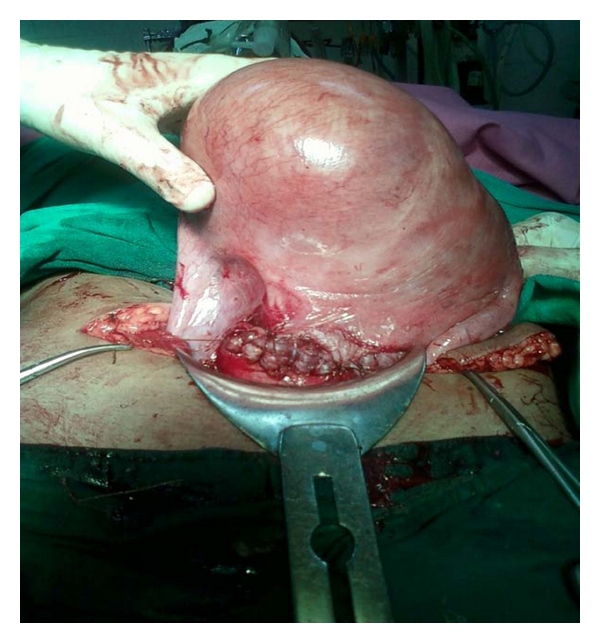
Detorsioned uterus with myoma after suturing.

A 25-years-old primigravida at 38 weeks of gestation reported to our hospital with severe intermittent and colicky abdominal pain of one-day duration. It started acutely but gradually increased in intensity involving all the abdomen. On examination, the patient was hemodynamically stable and afebrile. The uterus was term size and tender on palpation. A large mass of 10 × 10 cms was palpated on its right upper part. On vaginal examination os was 1 cm dilated and 30% effaced. Nonstress test (NST) was reactive on admission. Hemogram was normal and ultrasonography (USG) showed a single live intrauterine fetus with cephalic presentation with an intramural fibroid 10 × 10 cms on the right fundal region. Magnetic resonance imaging (MRI) confirmed the findings but torsion was not suspected since the classical sign on MRI was not seen as the films were not taken at the level of the vagina. Carneous degeneration of the fibroid or abruption placentae was suspected. As pain increased and fetal bradycardia of 90 bpm was there, patient underwent emergency cesarean under spinal anaesthesia. Abdomen was opened by pfannenstiel incision. On entering the abdominal cavity the left round ligament, ovary, and fallopian tubes were rotated to right and with manipulation that came into the view (Figure 1). As it was not possible to perform detorsion of the gravid uterus by exteriorization by pfannenstiel incision it was converted to vertical incision. Uterus was derotated by exteriorizing the myoma and the uterus out of the abdominal cavity. Once the torsion was corrected, lower segment cesarean was carried out. Alive female baby of 3 kgs weight was delivered. Uterus was closed in 2 layers and put back into abdomen (Figure 2). Abdomen was closed. Patient recovered well and was discharged on 5th postoperation day.

## 3. Discussion

Uterine torsion is defined as rotation of the uterus of more than 45 degrees on its long axis. It can range from 60–720 degrees. There is dextrorotation in two-third and levorotation in one-third of cases. The exact etiology is not known. Piot et al. [1], Jensen [2], Wilson et al. [3] have extensively reviewed the reports of torsion of gravid uterus. According to Wilson et al. [3] most of cases had normal anatomy that is unexplained torsion, where as according to Piot et al. [1] 31.8% had uterine myomata, 14.9% uterine anomalies especially bicornuate uterus, 8.4% had pelvic adhesions, 7% had ovarian cysts, 4.6% had abnormal presentation and fetal anomalies, 2.8% abnormalities of spine and pelvis, no discoverable causes in the rest of the cases. It is possible that cases without risk factors can be underreported. Other causes have been reported. Salani et al. [4] reported a case where ECV (external cephalic version) caused uterine torsion. They recommended to add torsion as one of complications of ECV. Duplantier et al. [5] reported only a case of torsion due to maternal trauma. Achanna et al. [6] have reported a case of torsion in uterus didelphys due to abdominal massage during labor by traditional birth attendants. According to Jensen [2] this condition can occur in all age groups, all parity, and all stages of pregnancy. Most cases are similar to our case with abdominal pain and tenderness and diagnosed only at laparotomy. They may present with birth obstruction, vaginal bleeding, shock, urinary and intestinal symptoms. High degree of suspicion is needed to diagnose this condition antenatally. Gule et al. [7] used modification of placental site compared to previous scan on USG (ultrasonography) and abnormal position of ovarian vessels across uterus on doppler to diagnose torsion. Change of position of fibroid can also be used to diagnose torsion on USG. Nicholson et al. [8] suggested X-shaped configuration of upper vagina on MRI (magnetic resonance imaging) as a sign to diagnose torsion. This is based upon the fact that vagina ia normally seen on MRI as an H-shaped structure, but with torsion of the uterus and upper vagina, the vagina appears as an X-shaped structure. Management requires emergency laparotomy. At term, uterus is derotated and LSCS (lower segment cesarean section) is done. If derotation is not possible, posterior low transverse incision is given [9]. In such cases elective section is advised in next pregnancy as risk of rupture is not known [3]. In difficult cases there are reports of myomectomy and posterior vertical section [10]. In all above methods it is important to delineate proper anatomy to prevent injury to major vessels and organs. In mid trimester uterus can be derotated and pelvic pathology causing torsion removed like myomectomy and ovarian cystectomy. To prevent recurrent torsion some have advocated plication of round ligament [11]. Mustafa et al. [12] have reported a case of plication of uterosacrals to prevent recurrent torsion. Jensen [2] has reported 13% perinatal mortality. There are no reported cases of maternal death after 1960 [7]. As the clinical presentation of torsion of the gravid uterus is vague, by knowing the risk factors we can suspect the condition and confirm it by MRI. This will lead to the better management of the condition with good maternal and fetal outcome.
